# Complete Genome Sequence of the Nonheterocystous Cyanobacterium *Pseudanabaena* sp. ABRG5-3

**DOI:** 10.1128/genomeA.01608-17

**Published:** 2018-02-08

**Authors:** Naoyuki Tajima, Yu Kanesaki, Shusei Sato, Hirofumi Yoshikawa, Fumito Maruyama, Ken Kurokawa, Hiroyuki Ohta, Tomoyasu Nishizawa, Munehiko Asayama, Naoki Sato

**Affiliations:** a Department of Life Sciences, Graduate School of Arts and Sciences, University of Tokyo, Tokyo, Japan; b NODAI Genome Research Center, Tokyo University of Agriculture, Tokyo, Japan; c Department of Environmental Life Sciences, Graduate School of Life Sciences, Tohoku University, Sendai, Japan; d Department of Bioscience, Tokyo University of Agriculture, Tokyo, Japan; e Section of Microbiology, Graduate School of Medicine and Faculty of Medicine, Kyoto University, Kyoto, Japan; f Department of Biological Information, Tokyo Institute of Technology, Yokohama, Japan; g Center for Biological Resources and Informatics, Tokyo Institute of Technology, Yokohama, Japan; h College of Agriculture, Ibaraki University, Ibaraki, Japan

## Abstract

We report here the complete sequences of the main genome (4.8 Mb) and seven plasmids of the semifilamentous, nonheterocystous cyanobacterium *Pseudanabaena* sp. ABRG5-3, a strain isolated from a pond in Japan. These data are expected to enhance our understanding of the *Pseudanabaena* subclade near the root of cyanobacterial diversity.

## GENOME ANNOUNCEMENT

Cyanobacteria comprise a group of oxygen-producing prokaryotes that engendered the initial oxidized atmosphere on the Earth, enabling the proliferation of heterotrophs and ultimately the emergence of large animals. Plastids of algae and plants are also likely to originate from a cyanobacterial endosymbiont (1). The genomic data of 54 strains of cyanobacteria, including recently sequenced ones (2), revealed two major clades: one with subclades C, D, and E comprising *Prochlorococcus*, *Synechococcus*, and *Leptolyngbya* spp., and another with subclades A and B comprising *Synechocystis*, *Anabaena*, *Cyanothece*, and many commonly studied species. *Gloeobacter* spp. diverged the earliest from the root, and there were three early branching subclades: subclade G comprising the unicellular Yellowstone strains, subclade F comprising the filamentous *Pseudanabaena* spp., and subclade E comprising unicellular strains of *Thermosynechococcus* and *Acaryochloris* spp., which have been extensively used for studies on photosynthesis. Ponse-Toledo et al. (1) identified that *Gloeomargarita lithophora*, which diverged between subclades F and E, was the closest sister to the primary plastids (plastids originating from the primary endosymbiosis), and *Pseudanabaena* spp. are likely to possess the same characteristics just before endosymbiosis. Although several strains of *Pseudanabaena* in subclade F have been sequenced, it is worth analyzing another strain (originally called *Limnothrix*), ABRG5-3 (3, 4), which is a semifilamentous, nonheterocystous cyanobacterium isolated from a pond in Japan; the genome sequence data are expected to enrich our understanding of the basal groups of cyanobacteria. We also found that the filaments of this strain showed rapid movement in bundles.

The cells of *Pseudanabaena* sp. ABRG5-3 were grown photoautotrophically in BG-11 medium (5). Total DNA was extracted by treatment with proteinase K and sodium *N*-dodecanoylsarcosinate, and purified by CsCl density gradient centrifugation as described previously (6). Purified DNA was subjected to sequencing with the 454 FLX+ genome sequencer (Roche Diagnostics, Indianapolis, IN, USA) and the Genome Analyzer II (Illumina, San Diego, CA, USA). Total genome data were assembled with Newbler version 2.5p1 into 290 contigs (average length = 19,331 bp), having an average read depth of 24.0. Gap regions were amplified by PCR and sequenced by the sequencing service of FASMAC Co. Ltd. (Atsugi, Japan). Finally, all gaps were completely filled. Open reading frames were detected by MetaGeneAnnotator (7), and tRNAs were estimated with tRNAscan-SE (8). rRNAs and noncoding RNAs were identified by a homology search with BLASTN (9) against known cyanobacterial genomes.

The main genome was a circular molecule of 4,796,642 bp (43.2% GC) and encoded 4,317 proteins, 52 tRNAs, 9 rRNAs (three sets of *rrs*, *rrl*, and *rrf* clusters), and 5 snRNAs. About one-third of the total proteins were hypothetical. We found gas vesicle proteins involved in floating and type IV pili proteins involved in twitching motility. We also detected 88 transposases. There were seven circular plasmids ranging from 12 kbp to 214 kbp. Plasmid ABRG53d contained a large cluster of nitrogen-fixing genes, which was similar to that encoded in the main genome of the nonheterocystous cyanobacterium *Leptolyngbya boryana* dg5 (10). Plasmids ABRG53a and ABRG53b encoded many homologous but hypothetical proteins.

Detailed genome and phylogenetic analyses are in progress.

### Accession number(s).

The complete genome sequences of the main genome and all seven plasmids were deposited in DDBJ/EMBL/GenBank under the accession numbers listed in Table 1. The versions described in this paper are the first version.

**TABLE 1:** Basic information for the genomes of *Pseudanabaena* sp. ABRG5-3

Genome	GenBank accession no.	Genome size (bp)
Main genome	AP017560	4,796,642
Plasmid ABRG53a	AP017561	214,764
Plasmid ABRG53b	AP017562	152,084
Plasmid ABRG53c	AP017563	134,662
Plasmid ABRG53d	AP017564	125,503
Plasmid ABRG53e	AP017565	111,135
Plasmid ABRG53f	AP017566	110,608
Plasmid ABRG53g	AP017567	12,574
